# Supplementary material for: A domain-knowledge modeling of hospital-acquired infection risk in Healthcare personnel from retrospective observational data: A case study for COVID-19
Source: PLoS One. 2022 Nov 21;17(11):e0272919. doi: 10.1371/journal.pone.0272919 (PMC9678325; doi:10.1371/journal.pone.0272919)
Supplement: S1 Table — (DOCX) [file pone.0272919.s001.docx]

**S1 Table. Characteristics of the selected features and their associated databases**

| **Features** | | **Values/Units** | **Notation** | **Characteristics** | **Data sources** |
| --- | --- | --- | --- | --- | --- |
| Time from symptom onset to hospitalization | | Days | $SOH_{time}$ | Secondary clinical attack rate is significantly high within the first 5 days from symptom onset | COVID-19 transmission dynamics data in Taiwan |
| Clinical severity of patients | | Discrete | $CS$ | Be classified into: Asymptomatic, Mild illness, Mild pneumonia, Severe pneumonia, and ARDS/sepsis |  |
| PPE sufficiency level | | % | $PPE_{SL}$ | To assess the sufficiency level, we used the answer of nurses and physicians to the question: “Does your hospital have adequate PPE for clinicians to treat the patients you have right now?”. In addition, Texas Health Center COVID-19 Survey Summary Report provided the Health Centers with an adequate supply of (PPE) for the next week. | 1. California COVID-19 Health Surveys: Data and Charts 2. Texas Health Center COVID-19 Survey Summary Report |
| Contact with others | | Score from 0 to 1 | $CO$ | The four physical job attributes help to determine the occupational-specific risk score:   - **Contact with others**: How much does this job require the worker to be in contact with others to perform it? - **Physical proximity**: To what extent does this job require the worker to perform tasks in close physical proximity to others? - **Exposure to disease/infection**: How often does this job require exposure to disease or infection? - **Working hours per week** | U.S. Department of Labor O*Net database |
| Physical proximity | |  | $PP$ |  |  |
| Exposure to disease/infection | |  | $EI$ |  |  |
| Working hours per week | |  | $N_{hours}$ |  |  |
| **Patient characteristics** | Age | Continuous | $Age$ | Age of HCP | Cross-sectional observational study of UK-based healthcare workers |
|  | Having Cancer | Binary | $Cancer$ | HCP’s comorbidities include cancer |  |
|  | Having respiratory disease | Binary | $Resp$ | HCP’s comorbidities include respiratory disease |  |
|  | Having obesity | Binary | $Obes$ | HCP’s comorbidities include obesity |  |
|  | Current or Ex-smoker within 1 year | Binary | $Smoker$ | HCP is a current smoker or ex-smoker within one year |  |
| **Work details** | Allied health professionals | Binary | $Allied\_prof$ | HCP is a current smoker or ex-smoker within one year |  |
|  | Dentists and dental staffs | Binary | $Dental\_staff$ | HCP is a dentist or a dental staff |  |
|  | Doctors | Binary | $Doctor$ | HCP is a doctor |  |
|  | Use public transport | Binary | $Pub\_trans$ | HCP uses public transport to travel to work |  |
| **Workplace exposure** | Regular clinical contact | Discrete | $C\_contact$ | Having Regular clinical contact with suspected or confirmed COVID-19 patients |  |
|  | Regular exposure to AGPs | Discrete | $AGP$ | Having Regular exposure to aerosol generating procedures (AGPs) performed in suspected or confirmed COVID-19 patients |  |
| **PPE usage** | Sufficient training in PPE use | Binary | $PPE\_train$ | Having Sufficient training in PPE use before handling patients |  |
|  | Lacked access to PPE | Binary | $Lacked\_PPE$ | Lacked access to PPE items for clinical contact with suspected or confirmed COVID-19 patients |  |
|  | Clinical contact without adequate PPE | Discrete | $Cont\_wo\_PPE$ | Be classified into never, rarely, sometimes, often, always |  |
|  | Used improvised PPE | Binary | $Imp\_PPE$ | HCP has used improvised (customized) PPE |  |
