# Supplementary material for: A domain-knowledge modeling of hospital-acquired infection risk in Healthcare personnel from retrospective observational data: A case study for COVID-19
Source: PLoS One. 2022 Nov 21;17(11):e0272919. doi: 10.1371/journal.pone.0272919 (PMC9678325; doi:10.1371/journal.pone.0272919)
Supplement: S1 File — (DOCX) [file pone.0272919.s002.docx]

**S1 File. The expectation and variance of the potential individual risk.**

Here, we assume that all contraction trials are independent within and between infection groups. The expected potential infection risk is estimated as:

$$E\left[ PIR_{i,j}^{\left( t_{1}:t_{2} \right)} \right]=E\left[ \sum_{m=1}^{\left| \boldsymbol{C}^{\left( \cdot\right)} \right|} \prod_{r=1}^{m-1} \left( 1-p_{X\left( r \right),k\left( r \right)\to j}^{\left( \cdot\right)} \right)p_{X\left( m \right),k\left( m \right)\to j}^{\left( \cdot\right)} \right]$$

$$=\sum_{m=1}^{\left| \boldsymbol{C}^{\left( \cdot\right)} \right|} \left[ \prod_{r=1}^{m-1} \left( 1-Ep_{X\left( r \right),k\left( r \right)\to j}^{\left( \cdot\right)} \right)Ep_{X\left( m \right),k\left( m \right)\to j}^{\left( \cdot\right)} \right]$$

Let’s assume that the viral transmission from a person $k$ to an HCP $j$ probability $p_{X,k\to j}^{\left( \cdot\right)} \sim Beta\left( \alpha_{X,k},\beta_{X,k} \right)$, where $X\in\left\{ S,E,I,HW \right\}$, because it is a canonical conjugate prior to the binomial distribution and a common prior for expert elicitation when limited data are available [1]. When more data are accumulated, the likelihood term would dominate prior distribution. Using the properties of beta distribution gives:

$$E\left[ PIR_{i,j}^{\left( t_{1}:t_{2} \right)} \right]=\sum_{m=1}^{\left| \boldsymbol{C}^{\left( \cdot\right)} \right|} \prod_{r=1}^{m-1} \left( \frac{\beta_{X\left( r \right),k\left( r \right)}}{\alpha_{X\left( r \right),k\left( r \right)}+\beta_{X\left( r \right),k\left( r \right)}} \right)\frac{\alpha_{X\left( m \right),k\left( m \right)}}{\alpha_{X\left( m \right),k\left( m \right)}+\beta_{X\left( m \right),k\left( m \right)}}$$

The variance $Var\left[ PIR_{i,j}^{\left( t_{1}:t_{2} \right)} \right]$ is:

$$Var\left[ PIR_{i,j}^{\left( t_{1}:t_{2} \right)} \right]=Var\left[ \sum_{m=1}^{\left| \boldsymbol{C}^{\left( \cdot\right)} \right|} \prod_{r=1}^{m-1} \left( 1-p_{X\left( r \right),k\left( r \right)\to j}^{\left( \cdot\right)} \right)p_{X\left( m \right),k\left( m \right)\to j}^{\left( \cdot\right)} \right]$$

Let’s denote $X_{r}:=p_{X\left( r \right),k\left( r \right)\to j}^{\left( \cdot\right)}, Y:=p_{X\left( m \right),k\left( m \right)\to j}^{\left( \cdot\right)}$, and $Z_{m}:=\prod_{r=1}^{m-1} \left( 1-X_{r} \right)Y$. We can assume $X_{r},Y,$ and $Z_{m}$ are i.i.d., which gives $Cov\left( Z_{i},Z_{j} \right)=0 \forall i\neq j$, we have:

$$Var\left( \sum_{m=1}^{\left| \boldsymbol{C}^{\left( \cdot\right)} \right|} Z_{m} \right)=\sum_{m=1}^{\left| \boldsymbol{C}^{\left( \cdot\right)} \right|} Var\left( Z_{m} \right)+\sum_{i\neq j} Cov\left( Z_{i},Z_{j} \right)=\sum_{m=1}^{\left| \boldsymbol{C}^{\left( \cdot\right)} \right|} Var\left( Z_{m} \right)$$

Therefore, to estimate $Var\left( \sum_{m=1}^{\left| \boldsymbol{C}^{\left( \cdot\right)} \right|} Z_{m} \right)$, we calculate the variance $Var\left( Z_{m} \right)$ where $m=1,\ldots,\left| \boldsymbol{C}^{\left( \cdot\right)} \right|$:

$$Var\left( Z_{m} \right)=Var\left[ \prod_{r=1}^{m-1} \left( 1-X_{r} \right)Y \right]$$

Let’s assume that $X_{i},Y\perp X_{j}^{n} \forall i\neq j$ and $X_{i}^{n}\perp Y^{n}$ where $\perp$ means “statistical independence” $i,j=1,\ldots m-1$ and $n\geq0$, which implies:

- $E\left[ X_{r}Y \right]=E\left[ X_{r} \right]E\left[ Y \right]$.
- $E\left[ X_{r}Y^{2} \right]=E\left[ X_{r} \right]E\left[ Y^{2} \right]=E\left[ X_{r} \right]\left[ Var\left( Y \right)+E\left[ Y \right]^{2} \right]$
- $cov\left( Y,X_{r}Y \right)=E\left[ {X_{r}Y}^{2} \right]-E\left[ X_{r}Y \right]E\left[ Y \right]=E\left[ X_{r} \right]Var\left( Y \right)$
- $cov\left( X_{i}Y,X_{j}Y \right)=E\left[ {X_{i}X_{j}Y}^{2} \right]-E\left[ X_{i}Y \right]E\left[ X_{j}Y \right]=E\left[ X_{i} \right]E\left[ X_{j} \right]Var\left( Y \right)$ if $i\neq j$
- $cov\left( X_{i}Y,X_{i}X_{j}Y \right)=Var\left( X_{i} \right)E\left[ X_{j} \right]Var\left( Y \right)+Var\left( X_{i} \right)E\left[ X_{j} \right]E\left( Y \right)^{2}+E\left[ X_{i} \right]^{2}E\left[ X_{j} \right]Var\left( Y \right)$ if $i\neq j$
- $cov\left( Y,X_{i}X_{j}Y \right)=E\left[ X_{i} \right]E\left[ X_{j} \right]Var\left( Y \right)$

For $m=1$, we have:

$$Var\left( Z_{m} \right)=Var\left( Y \right)$$

For $m=2$:

$$Var\left( Z_{m} \right)=Var\left[ \left( 1-X_{1} \right)Y \right]=Var\left( Y-X_{1}Y \right)=Var\left( Y \right)+Var\left( X_{1}Y \right)-2cov\left( Y,X_{1}Y \right)=Var\left( Y \right)+Var\left( X_{1}Y \right)-2E\left[ X_{1} \right]Var\left( Y \right)$$

For $m=3$:

$Var\left( Z_{m} \right)=Var\left[ \left( 1-X_{1} \right)\left( 1-X_{2} \right)Y \right]=Var\left( Y-X_{1}Y-X_{2}Y+X_{1}X_{2}Y \right)=Var\left( Y \right)+Var\left( X_{1}Y \right) +Var\left( X_{2}Y \right)+Var\left( X_{1}X_{2}Y \right)-2cov\left( Y,X_{1}Y \right)-2cov\left( Y,X_{2}Y \right)+2cov\left( Y,X_{1}X_{2}Y \right)+2cov\left( X_{1}Y,X_{2}Y \right)-2cov\left( X_{1}Y,X_{1}X_{2}Y \right)-2cov\left( X_{2}Y,X_{1}X_{2}Y \right)=Var\left( Y \right)+Var\left( X_{1}Y \right) +Var\left( X_{2}Y \right)+Var\left( X_{1}X_{2}Y \right)-2E\left[ X_{1} \right]Var\left( Y \right)-2E\left[ X_{2} \right]Var\left( Y \right)+2E\left[ X_{1} \right]E\left[ X_{2} \right]Var\left( Y \right)+E\left[ X_{1} \right]E\left[ X_{2} \right]Var\left( Y \right)-2\{Var\left( X_{1} \right)E\left[ X_{2} \right]Var\left( Y \right)+Var\left( X_{1} \right)E\left[ X_{2} \right]E\left( Y \right)^{2}+E\left[ X_{1} \right]^{2}E\left[ X_{2} \right]Var\left( Y \right)\}10-2\{Var\left( X_{2} \right)E\left[ X_{1} \right]Var\left( Y \right)$+ $Var\left( X_{2} \right)E\left[ X_{1} \right]E\left( Y \right)^{2}+E\left[ X_{2} \right]^{2}E\left[ X_{1} \right]Var\left( Y \right)\}$

Therefore, the general formula for $Var\left( Z_{m} \right)$ is complicated to derive and we used simulation-based methods to estimate the variance of $Z_{m}$ in our study.

**References**

1. Chaloner, K.M. and G.T. Duncan, *Assessment of a beta prior distribution: PM elicitation.* Journal of the Royal Statistical Society: Series D (The Statistician), 1983. **32**(1-2): p. 174-180.
