# Supplementary material for: A domain-knowledge modeling of hospital-acquired infection risk in Healthcare personnel from retrospective observational data: A case study for COVID-19
Source: PLoS One. 2022 Nov 21;17(11):e0272919. doi: 10.1371/journal.pone.0272919 (PMC9678325; doi:10.1371/journal.pone.0272919)
Supplement: Attachment — Submitted filename: Revision - Huynh et al.pdf [file pone.0272919.s003.pdf]

The submitted manuscript entitled *"A Domain-knowledge Modeling of Hospital-acquired Infection Risk in Healthcare Personnel from Retrospective Observational Data: A Case Study for COVID-19"* by Huynh et al. aims to develop infection risk models at both individual and population levels. The authors explore traditional SEIR dynamics to build an individual risk model and a Bayesian framework for the population indicator. The scope of the paper is well defined. I have only a shortlist of minor revisions that I would like to see addressed in a revised manuscript version.

Suggested revisions

- The abstract lacks a definition of "PPE"

- The abstract section results are populated with numbers without any explanation or context. It is hard to tell if the results are good or bad from the numbers only. Please refine the abstract to reflect the main takeaway points for a broader audience.

- Introduction: "*there has been an increasing hospital outbreaks"* → there has been an increasing number of hospital outbreaks.

- Introduction: "*Quantitative models have been used as an alternative to mathematical models."* I have the impression that Quantitative and mathematical models are synonyms. Perhaps the authors could use the alternative form: *Other quantitative approaches have been used…*

- Introduction: "*Section 2 discusses ~~about~~ the model formulation and model validation"*

- Introduction: I missed a review of modeling studies for nosocomial HCP infections at both individual and population levels. Since the paper is devoted to those two different problems, that could be reflected in the introduction. Instead, the authors briefly describe some previous work without that explicit distinction.

- Introduction: *To overcome the above research gaps, this paper proposes a probabilistic domain-knowledge model*

The term ``domain-knowledge'' appears throughout the manuscript. I wonder if there is a precise definition for the term in the context of this study (or references) that could be included in the introduction.

-Materials and Methods: "…(1) *an individual-level infection risk model that quantifies the risk of infection of an HCP… (2) a population-level infection risk indicator model that estimates the infection risk under working conditions at a medical facility"*

Odd sentence construction. I suggest: "(1) an individual-level infection risk model for HCP and (2) a population-level model that estimates the infection risk under working conditions… "

- Materials and methods, Section 2.1: In Eq. 4, the first equation is substituted by a summation indexed by m, and new variables h_m appear. Perhaps this is a standard calculation and I am missing some elementary steps, but it was unclear how the breakdown of h into h_m is done. Please clarify.

- Materials and methods, Section 2.1: "*where τ_r is the length of the close contact with person $k(m)$*"

It seems it should be τ_m instead of τ_r

- Materials and methods, Section 2.1:

The order in which the individual model is presented seems counterintuitive. After reading it carefully, I understood that the first step would be to calculate the probabilities $P_{X(r),k(r)\to j}$ from the logistic regression with the covariate data, and then the risk indicator could be estimated. The authors leave the logistic regression as the last step, which is confusing. Please consider addressing this point to improve the text clarity.

- Materials and Methods, Section 2.2: "*We denote $f(\cdot)$ as the abbreviated notation for the function of $PIR_{i,j}$ $(\cdot)$ and $\mathbf{F}$ in **Eq. (9)***"

It is unclear the role of Eq 9 in this sentence and the authors should avoid mentioning an equation before it appears in the text. Please clarify.

- Materials and Methods, Section 2.2: *"the population risk PER_i **(·)** is estimated using a Bayesian network…"*

The variable PER_i $(\cdot)$ has not been defined. I assume the authors meant PIR_i $(\cdot)$ instead

- Results: Caption of Fig. 3 "*Three data tips at P_{low} = {0.05, 0.2, 0.5} were"*

It seems an incomplete sentence.

- Results: Sections 3.2.1 are and 3.2.2 are devoted to well-known contributing factors and related work on HCP infection risk for COVID-19. Both sections do not contain results and should be part of the introduction or discussion.

- Results. Section 3.2.6: In Eq. 10, what is the value of the scaling parameter Φ?

- Results. Section 3.2.6: If $\hat{p}_{IC}$ $(t_1{:}t_2)$ is ORS/ max{ORS}, then it is unclear why Eq 6 was mentioned in the Materials and Methods section. Please clarify or omit any equation that is not used in the results section.

- Discussion: It is good practice to introduce a first paragraph reviewing the main problem, what methods were developed, and the main takeaways of a study. Please consider adding such a paragraph.
