# Supplementary material for: A domain-knowledge modeling of hospital-acquired infection risk in Healthcare personnel from retrospective observational data: A case study for COVID-19
Source: PLoS One. 2022 Nov 21;17(11):e0272919. doi: 10.1371/journal.pone.0272919 (PMC9678325; doi:10.1371/journal.pone.0272919)
Supplement: Attachment — Submitted filename: PLOSONE_Reviewer_Response_June16.docx [file pone.0272919.s004.docx]

We greatly appreciate the reviewers for your complimentary comments and suggestions. The manuscript has been revised. Specifically, we have addressed the following issues:

- Clarify the modeling assumptions and discuss their impacts on the model performance.
- Improve the readability and clarity of the text, figures, and tables.
- Improve the presentation of the mathematical formulas and the organization of the manuscript.
- Make the data sets publicly available on GitHub.

The updated parts in the manuscript were shown by the blue text. The followings are our point-by-point responses:

**Reviewer: 1**

The study successfully investigates the infection risk on a Bayesian network model and the model is validated against U.S. Department of Labor O*Net database. Public health experts, engineers, and epidemiologists can use the modified model when selecting different measures to reduce the infection risk from various respiratory diseases indoors, allowing informed decisions concerning environmental control.

**Response**:

We thank the reviewer for the feedback and the appreciation of the paper merits.

**Reviewer: 2**

1) I am mostly concerned about two modeling assumptions. First, that infection confer no immunity to the HCP and second, that I don't see clearly in the model the removal of infected HCP (confirmed or suspected) from regular activities. More specifically, I think that these assumptions may lead to an overestimation of cases which may affect parameter estimation. Nonetheless, these assumptions may still be valid in considering the worst-case limiting scenario which is still an interesting and valid first approach. I understand that changing these assumptions would be best left to future works, but I still believe that the limitations imposed by these assumptions should be more carefully addressed in the revision.

**Response**: We thank the reviewer for pointing out the two modeling assumptions that may cause the confusion and overestimation of model parameters. We initially mention these two assumptions and we regret not to clearly describe them in the previous manuscript, in the revised version we have update accordingly.

Regarding the first one – *“infection confer no immunity to the HCP”*, we highlighted this statement in the revised manuscript that – *“**we do not assume that the recovered patients confer immunity to reinfection when being released from isolation”*. This statement can be further clarified that even the patients are fully recovered after getting contracted with COVID-19 (or any communicable viral diseases in general) and being released back to the population, they are still under the risk of reinfection with the same disease strain or other strains. Therefore, if an HCP were recovered from the disease, they might get reinfected; however, they still confer some degree of immunity from previous infection. Therefore, we can consider adding a new group of patients in our model called “reinfected patients”. Moreover, the same idea can be applied for the vaccinated population, in which people have vaccine-induced immunity. We also have added three sentences in the **DISCUSSION** section to clarify better the assumption and discuss over the future work related to this assumption.

In terms of the second one – *“I don't see clearly in the model the removal of infected HCP (confirmed or suspected) from regular activities”*. We have added a sentence to clarify this point in the **Subsection 2.2**. Specifically, we do not consider the removal for individual-level risk estimation model because we are interested in estimating the infection risk of HCP over a certain period. However, when it comes to population-level model, the infection risk will be considered for the whole medical facility or hospital; hence, the estimated individual-level risk will contribute to estimate the population-level risk over time (as shown in the **Equation 2.10** in the manuscript). When the working restriction policy is applied to a certain HCP, which forces him/her to be self-isolated at home, his/her individual risk will not be considered in that equation.

2) I also recommend the improvement of data availability. If data can be provided upon request, I do not understand why it cannot be made publicly available in an online repository such as github or gitlab. If not possible, please justify your decision.

**Response**: We thank the reviewer for the suggestion. We do not own the datasets, so we are only able to provide the links and references to the following datasets. It is publicly available at <https://github.com/PhatKHuynh/COVID-19-datasets> on GitHub.

3) Tables and figures are of sufficient quality, but their readability could be improved by reducing the use of abbreviations a little. Most importantly, I request that you improve the naming of covariates in the model, exposed in section 3.2.4 and Table 2. Resolution of figures should be improved for final manuscript.

**Response**: We appreciate the reviewer suggestion. We have revised the tables and figures to explain the used abbreviations and improve the quality. Furthermore, an extra column has been inserted in Table 2 to provide the description of the variables. In addition, the notations of the covariates have also been revised for the ease of understanding. The revision has been made in the revised manuscript.

4) Additionally, there are a couple of incorrect closures of parenthesis in the last paragraph of section 2; please correct these and verify carefully if such mistakes escaped our eyes elsewhere. It would be appreciated if line numbering was incorporated in the revision to facilitate the correction of such small problems for the final manuscript.

**Response**: We regret for the oversight. We have revised the mistakes of incorrect closure of parentheses and incorporated the line numbering in our revised manuscript.

**Reviewer: 3**

1) The abstract lacks a definition of “PPE’’.

- The abstract section results are populated with numbers without any explanation or context. It is hard to tell if the results are good or bad from the numbers only. Please refine the abstract to reflect the main takeaway points for a broader audience.

**Response**: We truly regret for the oversight. We have updated the definition of PPE in the abstract and revised the abstract content to make the summary of the results clearer and more understandable.

2) Introduction: “Quantitative models have been used as an alternative to mathematical models.” I have the impression that Quantitative and mathematical models are synonyms. Perhaps the authors could use the alternative form: Other quantitative approaches have been used…

- Introduction: “Section 2 discusses ~~about~~ the model formulation and model validation”

- Introduction: I missed a review of modeling studies for nosocomial HCP infections at both individual and population levels. Since the paper is devoted to those two different problems, that could be reflected in the introduction. Instead, the authors briefly describe some previous work without that explicit distinction.

- Introduction: To overcome the above research gaps, this paper proposes a probabilistic domain-knowledge model. The term ``domain-knowledge’’ appears throughout the manuscript. I wonder if there is a precise definition for the term in the context of this study (or references) that could be included in the introduction.

**Response**: We appreciate the reviewer for the detailed comments for the INTRODUCION section and apologize for the oversight. We address them point by point as follows:

- Comment: *“Quantitative models have been used as an alternative to mathematical models.”* I have the impression that Quantitative and mathematical models are synonyms. Perhaps the authors could use the alternative form: *“Other quantitative approaches have been used…”*
- We have changed the term “quantitative models” to “statistical models” to avoid the confusion with the mathematical epidemiological models in the revised manuscript.
- Comment: *“Section 2 discusses ~~about~~ the model formulation and model validation”*
- The grammar mistake has been corrected.
- Comment: I missed a review of modeling studies for nosocomial HCP infections at both individual and population levels. Since the paper is devoted to those two different problems, that could be reflected in the introduction. Instead, the authors briefly describe some previous work without that explicit distinction.
- We have updated the literature review paragraph related to modeling studies for HCP risk infection estimation at both individual and population levels and made a clear distinction between studies at both levels. It has been updated in the INTRODUCION section of the revised manuscript.
- Comment: To overcome the above research gaps, this paper proposes a probabilistic domain-knowledge model. The term “domain-knowledge” appears throughout the manuscript. I wonder if there is a precise definition for the term in the context of this study (or references) that could be included in the introduction.
- We agree that the term “domain-knowledge” is well-known in machine learning field but may not be familiar with epidemiological modelers. We have added some sentences to define the term “domain-knowledge” in the context of this study and provide relevant references in the last paragraph in the **INTRODUCTION** section in the revised manuscript.

3) Materials and Methods: “…(1) an individual-level infection risk model that quantifies the risk of infection of an HCP… (2) a population-level infection risk indicator model that estimates the infection risk under working conditions at a medical facility”. Odd sentence construction. I suggest: “(1) an individual-level infection risk model for HCP and (2) a population-level model that estimates the infection risk under working conditions…”

- Materials and methods, Section 2.1: In Eq. 4, the first equation is substituted by a summation indexed by $m$, and new variables $h_{m}$ appear. Perhaps this is a standard calculation, and I am missing some elementary steps, but it was unclear how the breakdown of $h$ into $h_{m}$ is done. Please clarify.

- Materials and methods, Section 2.1: “where $\tau_{r}$ is the length of the close contact with person $k\left( m \right)$”. It seems it should be $\tau_{m}$ instead of $\tau_{r}$.

**Response**: We appreciate the reviewer for the detailed comments for the MATERIALS AND METHODS section and apologize for the oversight. We address them point by point as follows:

- Comment: *“(1) an individual-level infection risk model that quantifies the risk of infection of an HCP… (2) a population-level infection risk indicator model that estimates the infection risk under working conditions at a medical facility”. Odd sentence construction. I suggest: “(1) an individual-level infection risk model for HCP and (2) a population-level model that estimates the infection risk under working conditions”*
- We have revised the sentence in our manuscript.
- Comment: **Section 2.1**: In **Eq. 4**, the first equation is substituted by a summation indexed by $m$, and new variables $h_{m}$ appear. Perhaps this is a standard calculation, and I am missing some elementary steps, but it was unclear how the breakdown of $h$ into $h_{m}$ is done. Please clarify.
- The grammar mistake has been corrected.
- Comment: **Section 2.1**: “where $\tau_{r}$ is the length of the close contact with person $k\left( m \right)$”. It seems it should be $\tau_{m}$ instead of $\tau_{r}$*”*
- We have revised the notation in the manuscript.

4) Materials and methods, **Section 2.1**: The order in which the individual model is presented seems counterintuitive. After reading it carefully, I understood that the first step would be to calculate the probabilities $P_{X\left( r \right),k\left( r \right)\to j}$ from the logistic regression with the covariate data, and then the risk indicator could be estimated. The authors leave the logistic regression as the last step, which is confusing. Please consider addressing this point to improve the text clarity.

- Materials and Methods, **Section 2.2**: “We denote $f(\cdot)$ as the abbreviated notation for the function of $PIR_{i,j}^{\left( . \right)}$, $(\cdot)$ and $\boldsymbol{F}$ in **Eq. (9)**”. It is unclear the role of **Eq 9** in this sentence and the authors should avoid mentioning an equation before it appears in the text. Please clarify.

- Materials and Methods, **Section 2.2**: “the population risk $PER_{i}^{\left( . \right)}$ is estimated using a Bayesian network…”. The variable $PER_{i}^{\left( . \right)}$ has not been defined. I assume the authors meant $PIR_{i}^{\left( . \right)}$ instead.

**Response**: We appreciate the reviewer for the detailed comments for the MATERIALS AND METHODS section and apologize for the oversight. We address them point by point as follows:

- Comment: **Section 2.1**: The order in which the individual model is presented seems counterintuitive. After reading it carefully, I understood that the first step would be to calculate the probabilities $P_{X\left( r \right),k\left( r \right)\to j}$ from the logistic regression with the covariate data, and then the risk indicator could be estimated. The authors leave the logistic regression as the last step, which is confusing. Please consider addressing this point to improve the text clarity.
- We have revised the section that presents the steps of the individual-level model, in which we rearrange the equations and content to improve the text clarity.
- Comment: Section 2.2: *“We denote* $f(\cdot)$ *as the abbreviated notation for the function of* $PIR_{i,j}^{\left( . \right)}$*,* $(\cdot)$ *and* $\boldsymbol{F}$ *in* ***Eq. (9)****”.* It is unclear the role of **Eq 9** in this sentence and the authors should avoid mentioning an equation before it appears in the text. Please clarify.
- We have revised the sentence and removed the reference of **Eq. (9)** before it appears.
- Comment: **Section 2.2**: *“the population risk* $PER_{i}^{\left( . \right)}$ *is estimated using a Bayesian network…”.* The variable $PER_{i}^{\left( . \right)}$ has not been defined. I assume the authors meant $PIR_{i}^{\left( . \right)}$ instead.
- We have revised the notation in the manuscript.

5) - Results: Caption of Fig. 3 “Three data tips at = {0.05, 0.2, 0.5} were”
   It seems an incomplete sentence.
   - Results: **Sections 3.2.1** are and **3.2.2** are devoted to well-known contributing factors and related work on HCP infection risk for COVID-19. Both sections do not contain results and should be part of the introduction or discussion.
   - Results. **Section 3.2.6**: In Eq. 10, what is the value of the scaling parameter $\Phi$?
   - Results. **Section 3.2.6**: if $PIR^{\left( t_{1}:t_{2} \right)}$ is $ORS/\max\left\{ ORS \right\}$, then it is unclear why **Eq 6** was mentioned in the Materials and Methods section. Please clarify or omit any equation that is not used in the results section.

**Response**: We appreciate the reviewer for the detailed comments for the RESULTS section and apologize for the oversight. We address them point by point as follows:

- Comment: Caption of **Fig. 3** *“Three data tips at = {0.05, 0.2, 0.5} were”.* It seems an incomplete sentence.
- We sorry for the oversight, we have revised the caption of **Fig. 3** in the manuscript.
- Comment: **Sections 3.2.1** and **3.2.2** are devoted to well-known contributing factors and related work on HCP infection risk for COVID-19. Both sections do not contain results and should be part of the introduction or discussion.
- We have changed **Section 3. RESULTS** to **Section 3. RESULTS AND COVID-19 CASE STUDY** and merged **Sections 3.2.1** and **3.2.2** to one common section called **Sections 3.2.1. Case study description.**
- Comment: In **Eq 10*,*** *what is the value of the scaling parameter* $\phi$*?*
- We have added a sentence to provide the selected value of $\phi$ in **Eq 10**. The value of $\phi$ is set to be 10 for the in the model validation of individual-level infection risk (**Section 3.2.6**)
- Comment: **Section 3.2.6**: If $PIR^{\left( t_{1}:t_{2} \right)}$ is $ORS/\max\left\{ ORS \right\}$, then it is unclear why **Eq 6** was mentioned in the **Materials and Methods** section. Please clarify or omit any equation that is not used in the **results** section.
- We have clarified the relationship between the **Eq 6** and the estimation of $PIR^{\left( t_{1}:t_{2} \right)}$ as $ORS/\max\left\{ ORS \right\}$ and updated in our manuscript. The formula $ORS/\max\left\{ ORS \right\}$ is used as a surrogate approach for approximating the transmission probability defined in **Eq. (6)** in the scenario of limited individual-level data.

6) - Discussion: It is good practice to introduce a first paragraph reviewing the main problem, what methods were developed, and the main takeaways of a study. Please consider adding such a paragraph.

**Response**: We thank the reviewer for the suggestion for the first paragraph of the **DISCUSSION section** to review the main problem, the proposed methods, and the key takeaways of the study. We have composed the paragraph and updated in the **DISCUSSION section** of the revised manuscript.
